# Mr. Best, on Uterine Rupture

**Published:** 1805-03-01

**Authors:** Henry Best

**Affiliations:** Thetford


### *To the Editors of the Medical and Physical Journal.*

GENTLEMEN,

UPON referring to all the volumes of the Medical and Physical Journal now extant, I do not find that any practitioner among your Correspondents has given a single paper on the subject of Uterine Rupture. If the following case and observations should be considered sufficiently important for insertion in that valuable work, they are very much at your service.

I am, &c.

*Thetford, Dec.* 30, 1804. HENRY BEST.

---

MONDAY the 29th of last October, about one o'clock in the morning, I was requested to give my attendance as accoucheur on Mrs. Ransom, the wife of a respectable farmer residing at Hockham, a village about eight miles from this town. Her full term of gestation had expired, and I was informed, that some time before my arrival, a copious evacuation had taken place from her bowels; that during a pain, the membranes had given way, and the discharge of the liquor amnii had been very considerable; from which time the pains had been so strong and frequent, that both herself and her attendants thought the labour was making rapid progress towards a favourable termination. Upon examination per vaginam, I found the os uteri very little dilated, and the child so high up I could not obtain the smallest knowledge of its situation; and although the pains continued strong, and returned at short intervals, it was not till the evening of this day that the uterine orifice would allow me to satisfy myself, as to the nature of the presentation, when, with some little difficulty, I passed my hand, and found the head entering the superior aperture of the pelvis.

During Monday night, and the forenoon of Tuesday, she betrayed a considerable degree of inquietude, was frequently changing her posture, sometimes lying upon one side side and sometimes upon the other, and every now and then out of bed, and walking about the room; but with a view of relieving these restless emotions she was prevailed upon to take a few drops of laudanum, which in some measure answered the desired purpose; the pains became suspended, and she enjoyed a few broken slumbers; but as soon as that medicine had ceased to act, the pains returned with their usual strength, and recurred at short regular intervals. From time to time she took a little gruel, and now and then a draught of small-beer, and frequently passed her urine. The os uteri became soft, yielding, and fully dilated. The labour made gradual progress, and at this period in no respect varied from common natural cases. By the continued and regular force of pain the head on Tuesday afternoon descended into the concavity of the os sacrum, and although the liquor amnii had been evacuated at so early a period, the state of the soft parts, the regular recurrence and strength of pain, the calmness and regularity of the pulse, and every other circumstance, promised a speedy and happy delivery. About six o'clock in the evening of this day, a servant entered the room and announced tea, when I withdrew with one of the attending ladies to another apartment, but was immediately followed by the nurse, who said "the pains had stopped all at at once," and that Mrs. R. wished to see me. Upon approaching the side of her bed, she exclaimed, "Oh Doctor! the child has slipped through to my side, and I am sure I am dying." Her respiration immediately became so quick and laborious, that she insisted upon being taken up, and was accordingly lifted out of bed; she walked a few steps, and then seated herself on the sfde of the bed, kept her hand to her side, and frequently repeated that she was worse and worse, and dying. Upon applying my hand to the abdomen, I distinctly discovered that some part of the child had bursted through the uterus; while vomiting, hiccuping, uterine hæmorrhage, cessation of labour pain, &c. but too clearly convinced me of the fatal catastrophe. Her respiration became more and more oppressed, and in a few minutes spasms took place, and death succeeded. In this melancholy case the symptoms of dissolution rushed on with such rapidity, and fixed her so irremoveably to the sitting posture, neither time nor opportunity allowed of any attempt to deliver the child. After an interval of thirty-six hours, I was permitted, in the presence of Mr. Mingay (with whom I am professionally connected) the nurse and another female, to open and inspect the body.

Upon making a division in the usual manner, the whole of the child with the placenta were found among the abdominal viscera, but the quantity of bloody serum contained in the cavity, together with the extreme putrid state of the parts, prevented me from ascertaining the precise situation and direction of the laceration in utero.

Upon examining the child, after removing it from the mother, it was not found to exhibit any appearance the least unusual.

Some time during the last month of Mrs. Ransom's pregnancy, I understood, that in taking the winch of the butter churn rather hastily from the servant, she received a violent blow upon the prominent part of her belly, which for some time occasioned considerable uneasiness; she mentioned this circumstance once or twice during her labour, but did not seem to attribute any ill consequence to the accident.

Mrs. R. was in the fortieth year of her age, of middling stature, and much disposed to obesity; but in general had enjoyed a good state of health. She was mother of eleven children, all now living; and as a proof there was no fault in the formation of the pelvis, she had never before experienced a difficult or even a tedious labour.

Among practitioners in Midwifery, I believe considerable opposition has prevailed in answer to the question, whether or not any attempt should be made to deliver a woman who has suffered a rupture of the uterus. The late celebrated Dr. Hunter is one among the number of those who have considered this dreadful accident as inevitably fatal both to the mother and child, and appeared to have thought an attempt to deliver, as answering no other purpose but that of increasing the sufferings of a fellow creature. In a case of this nature, quoted by Dr. A. Douglas, in which Dr. Denman was consulted, it appears that, at that period, the Doctor had no idea any woman could survive a rupture of the uterus, and therefore considered an attempt to extract the child, not only as hopeless, but likely to answer no other end but that of aggravating the sufferings of the patient. This celebrated and experienced practitioner, however, in the last edition of his excellent Treatise on the subject of Midwifery, appears to consider it our duty, "hopeless as the case must be, to pass the hand into the uterus, to turn and deliver the child by the feet, or with the forceps or vectis, or in any other manner the case will allow."

In the year 1789, the public was favored with a pamphlet, phlet, entitled, "Observations on the Rupture of the gravid Uterus, by Dr. A. Douglas." In that interesting Essay the Doctor records a case of the kind in question, in which he was consulted, and where the circumstances of the labour urged him to turn, and extract the child; and it appears, from the clearest evidence, the woman actually recovered; that the lacerated part of the uterus reunited; and that she afterwards had two children. That the case here alluded to was that of a rupture of the uterus, the Dr's statement proves beyond a possibility of doubt; and as a copy of the most interesting part of it may not be unacceptable to such of your readers who have not had the opportunity of perusing the original; and as, at the same time, it will prove, if there is a possibility of delivering a woman who has suffered a rupture of the uterus, that the case is not entirely destitute of hope, I shall take the liberty of subjoining it.

"By examining in the common way, I could distinguish nothing except a round movable substance, which I supposed to be the head of the child; but being able to reach it only with the points of my fingers, I could not determine with any certainty what it was. The woman appearing to be, in extremis, my mind was so wholly occupied by her danger, as to preclude all reasoning with respect to the cause. Immediate delivery seemed to promise the only chance of relief, which, though slender, I resolved to afford her, by turning the child. There was no difficulty in passing my hand; and the substance which I had supposed to be the child, fled before the tips of my fingers. By following it, I at last perceived my hand was in a cavity which in no sort resembled the uterus. I was then forcibly and painfully struck with the nature of the case; and on examining all round, could with certainty determine that my hand was in the cavity of the abdomen, the child lying on the fore part, on the back part the contracted uterus like an oblong ball, and the intestines hanging among my fingers. It is not easy to imagine how miserable I felt at this moment: No hope of doing good to the woman from the usual event of such cases; no possibility of consulting with any of my brethren; and a necessity of determining instantly how I was to proceed. I decided in favor of immediate delivery; my hand being in contact with the child, the feet easily to be found, and no possible advantage to be expected from delay. The death of the patient was what might reasonably be expected. I met with no obstruction to the turning and extraction, tion of the child, except in the passage of the head thro' the brim of the pelvis. While my hand was in search of the feet, I thought I could perceive that the placenta was likewise in the abdominal cavity, therefore expected that it would be easily brought away. But in this I was mistaken; for it had so clung to the intestines, that I was under the necessity of again introducing my hand, to detach it. This was not difficult, and afforded me an opportunity of being still better instructed in the nature of the injury. The uterus seemed to have ruptured transversely, on the lower and fore part, some distance above where the vagina is connected with it, and was more contracted in its size than I thought possible in the short space of time which had elapsed since the accident. The hæmorrhage was not greater than is usual in common labours."

It appears from the Doctor's diary, that the subject of the above case was harrassed with many very distressing and threatening symptoms during the first fortnight; but that at the end of forty-five days she was so far recovered, that she walked from Clare Market to the Doctor's house in Bedford Square, without much inconvenience.

Although the experience of the past and of the present time has rendered cases of this nature almost hopeless, yet in the opinion of Dr. Denman, as well as that of Dr. Andrew Douglas, the above remarkable instance of recovery is sufficient to authorize every practitioner in future, where the case will possibly admit of it, to endeavour to deliver the patient, "and bad as her chance certainly would be, to be strenuous in using all the means which art dictates to extricate her from her danger."

It appears from the authority of Dr. Douglass, that rupture of the gravid uterus has happened more frequently than practitioners in general may have been aware of. He asserts that twenty cases, in which the fact was demonstrated by dissection, had occurred within twenty years in London, and adds, that a considerable proportion of the deaths which have happened suddenly in time of labour, or soon after delivery, may probably have been in consequence of a laceration of the womb or its appendages.

